# Supplementary figures and images for: Comprehensive Functional Analysis of the bZIP Family in Bletilla striata Reveals That BsbZIP13 Could Respond to Multiple Abiotic Stresses
Source: Int J Mol Sci. 2023 Oct 15;24(20):15202. doi: 10.3390/ijms242015202 (PMC10607107; doi:10.3390/ijms242015202)

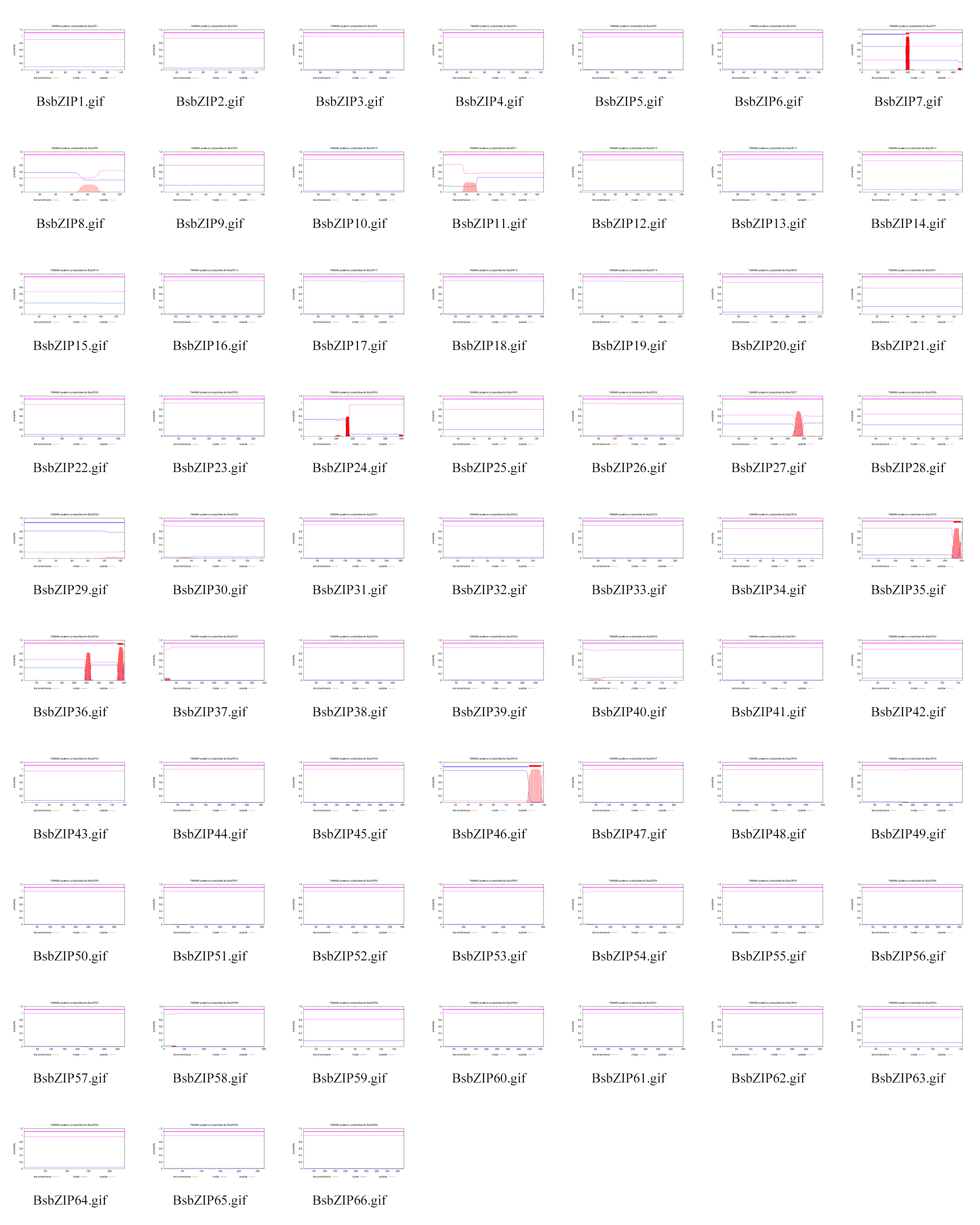

Supplement: Supplementary file 1 [file ijms-24-15202-s001.zip › Supplemental Files/Fig. S1.jpg]

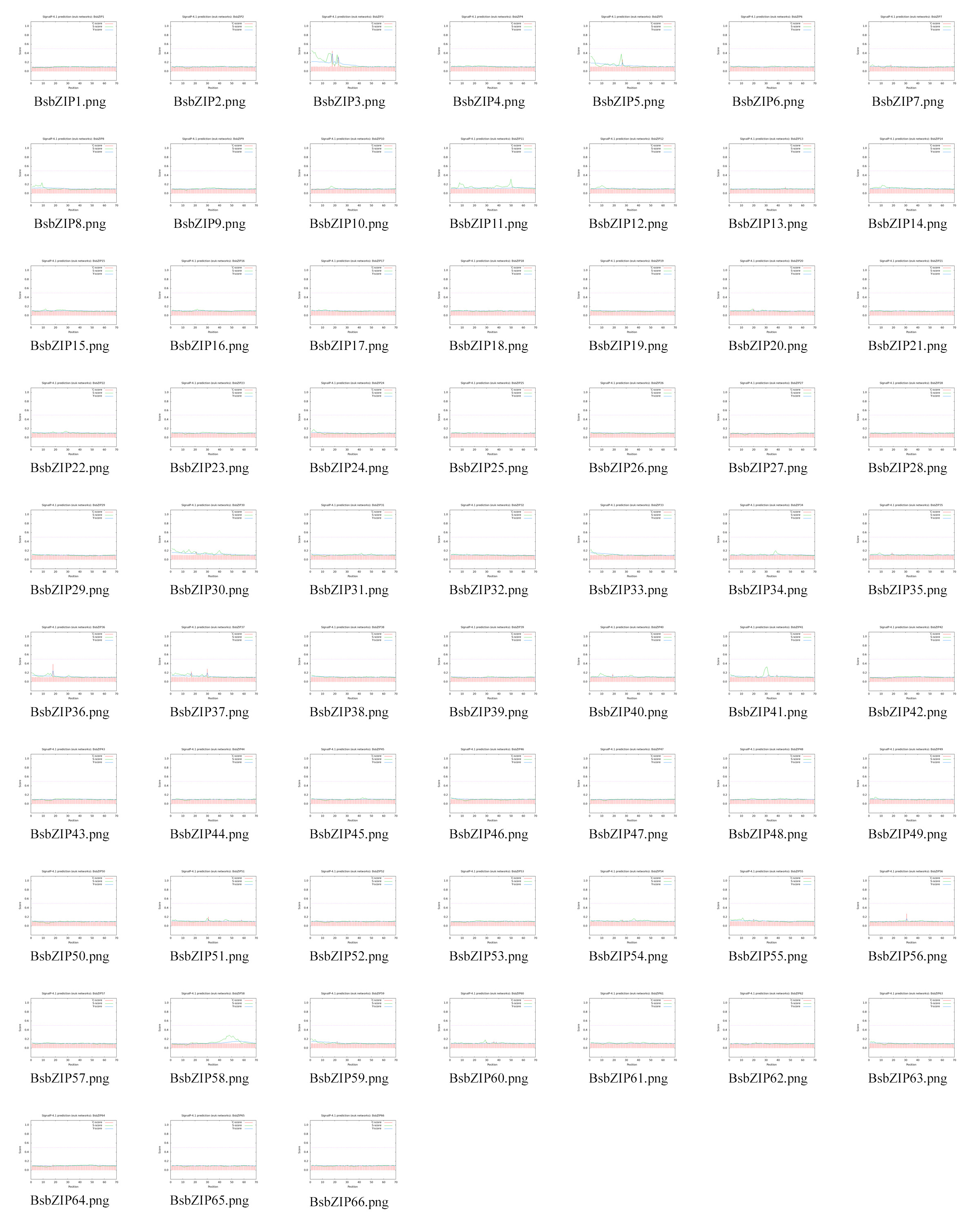

Supplement: Supplementary file 1 [file ijms-24-15202-s001.zip › Supplemental Files/Fig. S2.jpg]

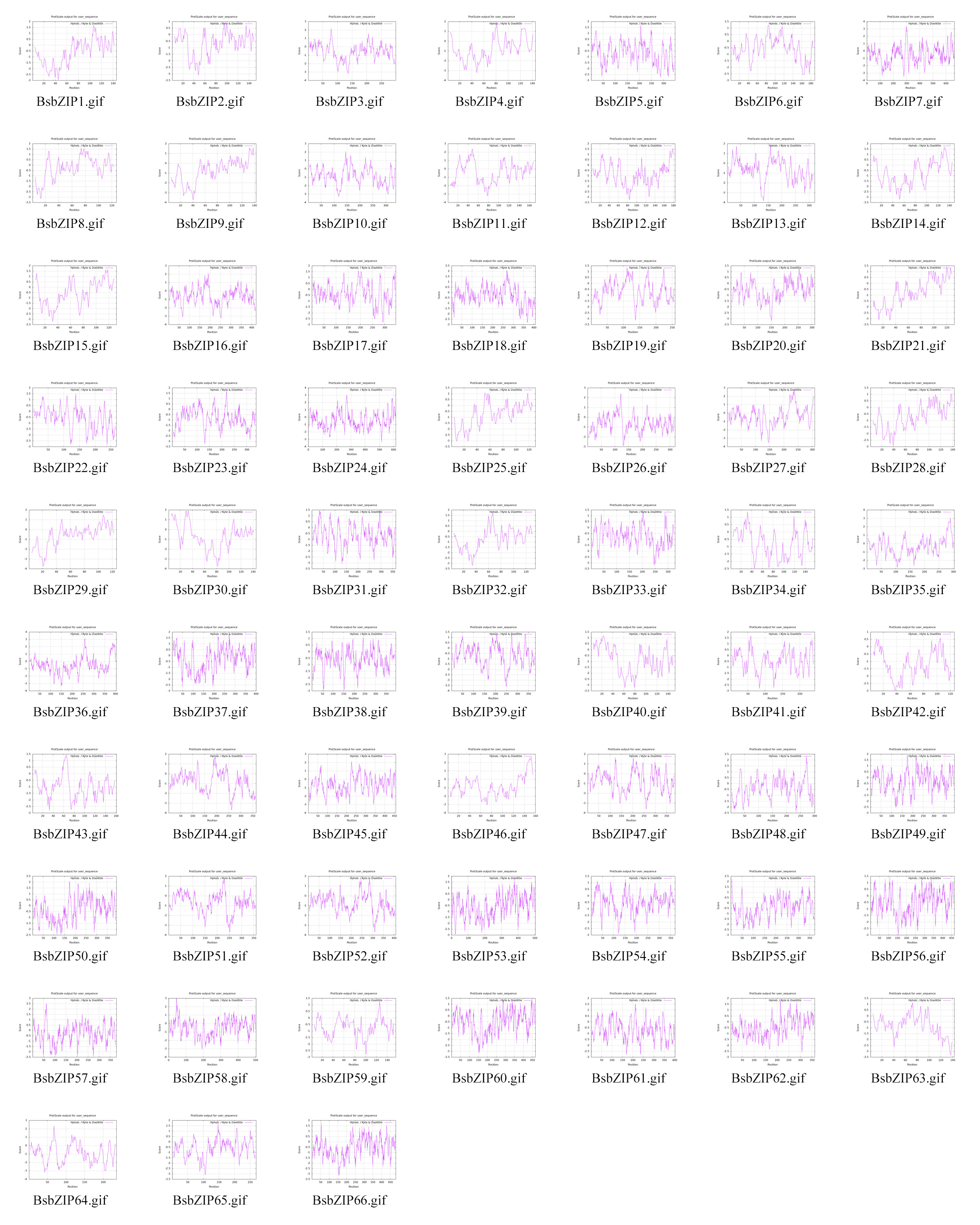

Supplement: Supplementary file 1 [file ijms-24-15202-s001.zip › Supplemental Files/Fig. S3.jpg]

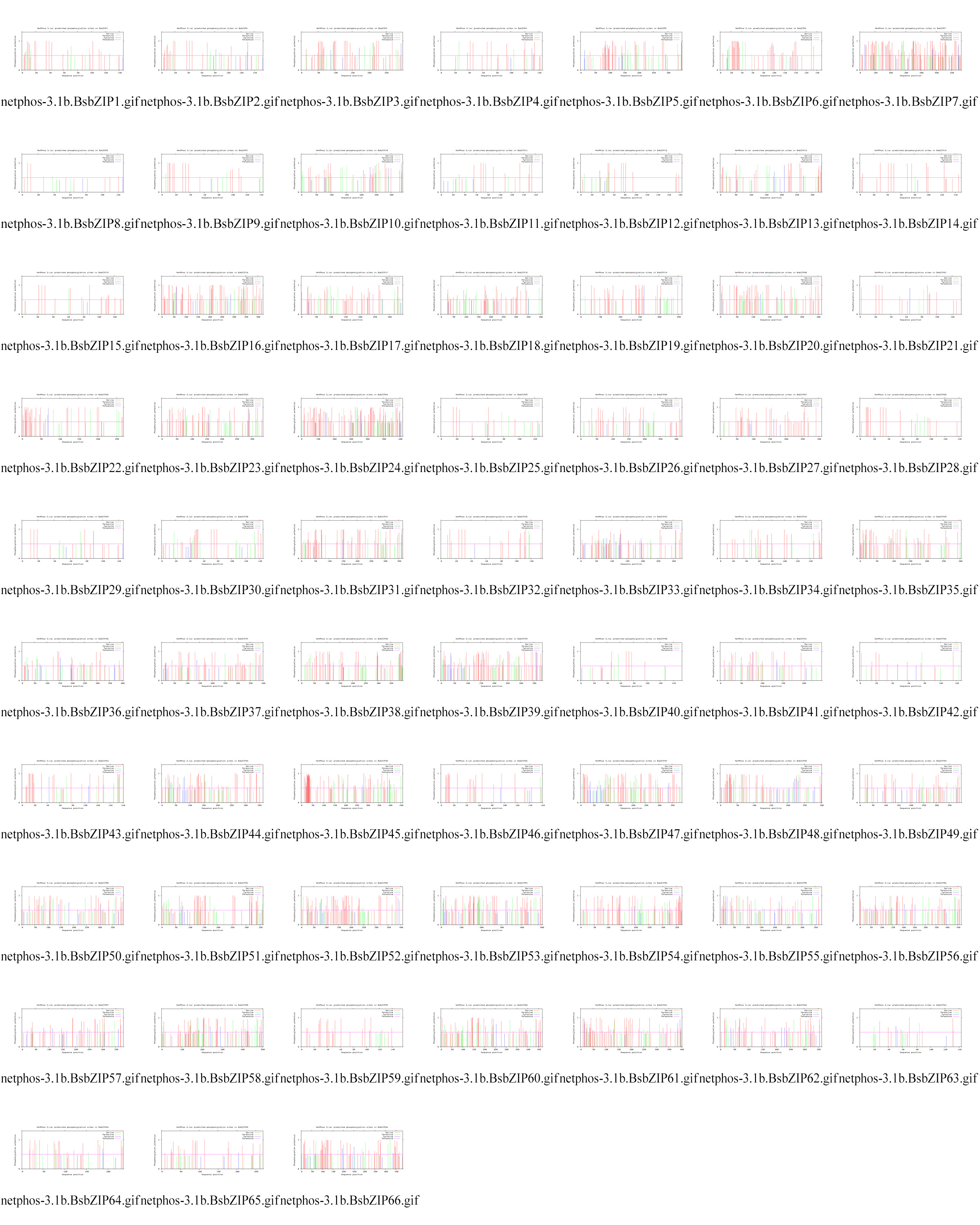

Supplement: Supplementary file 1 [file ijms-24-15202-s001.zip › Supplemental Files/Fig. S4.jpg]

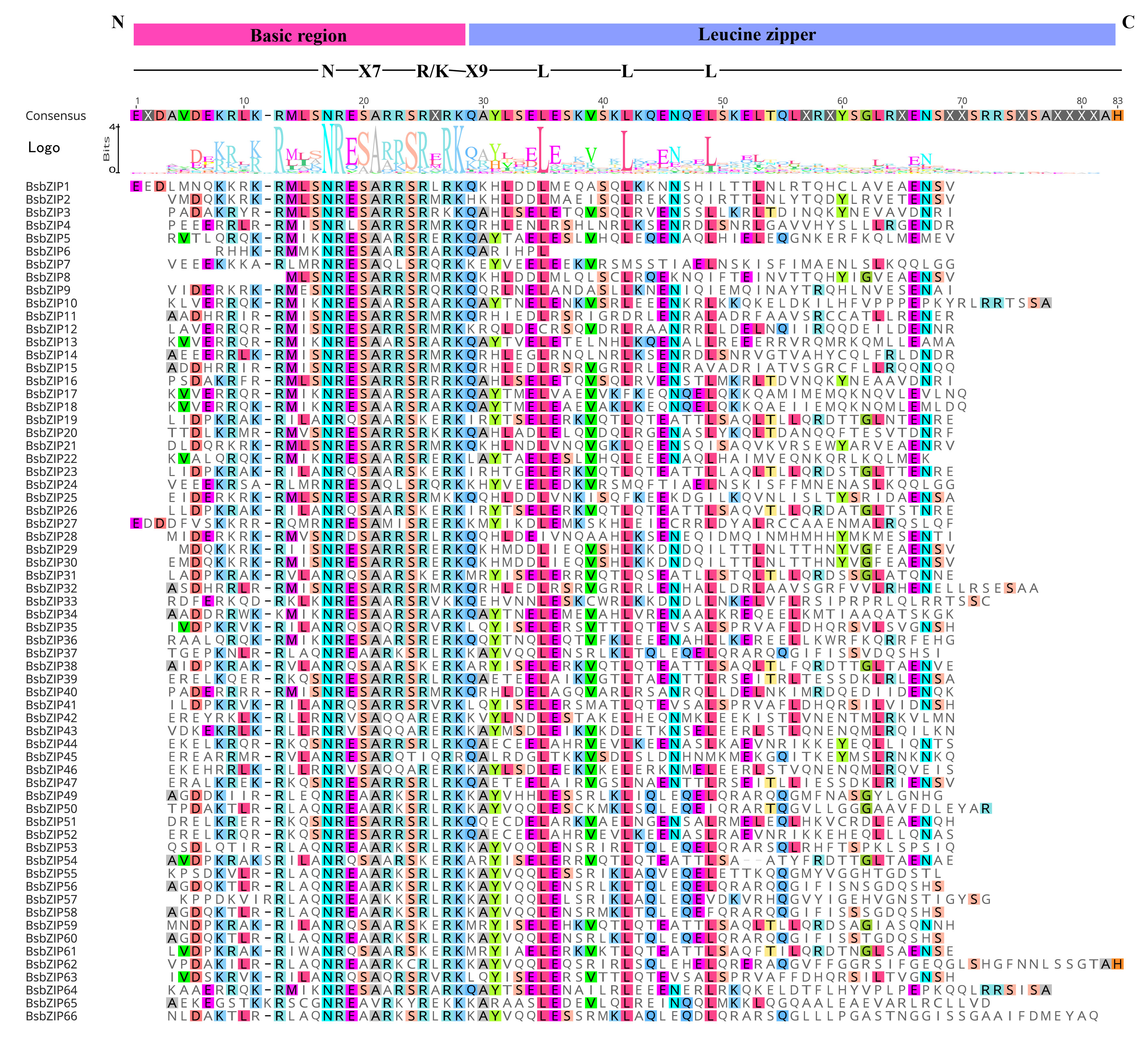

Supplement: Supplementary file 1 [file ijms-24-15202-s001.zip › Supplemental Files/Fig. S5.jpg]

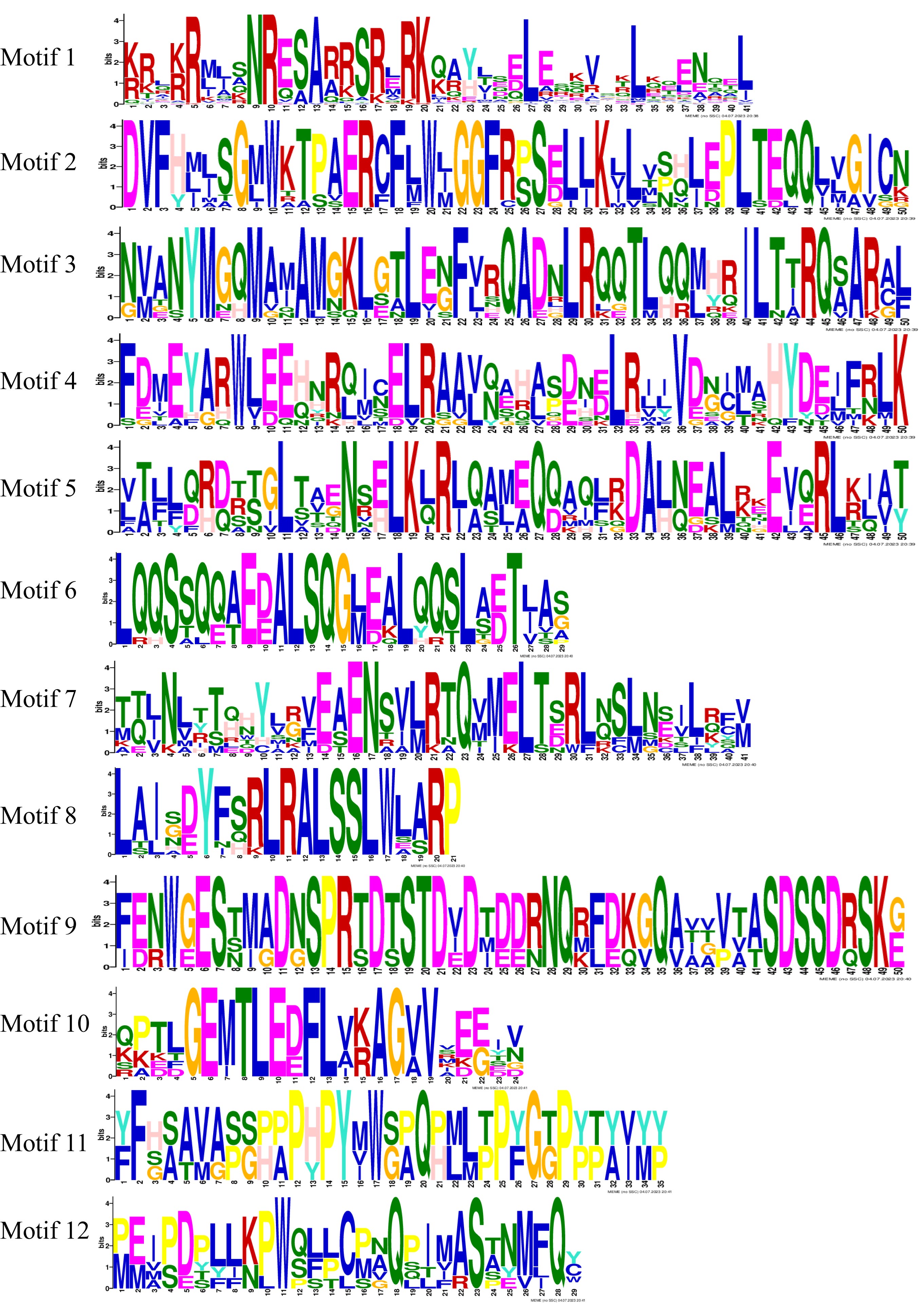

Supplement: Supplementary file 1 [file ijms-24-15202-s001.zip › Supplemental Files/Fig. S6.jpg]
